# A computational stochastic procedure for solving the epidemic breathing transmission system

**DOI:** 10.1038/s41598-023-43324-2

**Published:** 2023-09-27

**Authors:** Najah AbuAli, Muhammad Bilal Khan, Zulqurnain Sabir

**Affiliations:** 1 College of Information Technology, UAE University, P. O. Box 15551, Al Ain, UAE; 2 Department of Mathematical Sciences, UAE University, P. O. Box 15551, Al Ain, UAE; 3 Department of Computer Science and Mathematics, Lebanese American University, Beirut, Lebanon

**Keywords:** Computational biology and bioinformatics, Engineering, Mathematics and computing

## Abstract

This work provides numerical simulations of the nonlinear breathing transmission epidemic system using the proposed stochastic scale conjugate gradient neural networks (SCGGNNs) procedure. The mathematical model categorizes the breathing transmission epidemic model into four dynamics based on a nonlinear stiff ordinary differential system: susceptible, exposed, infected, and recovered. Three different cases of the model are taken and numerically presented by applying the stochastic SCGGNNs. An activation function ‘log-sigmoid’ uses twenty neurons in the hidden layers. The precision of SCGGNNs is obtained by comparing the proposed and database solutions. While the negligible absolute error is performed around 10^–06^ to 10^–07^, it enhances the accuracy of the scheme. The obtained results of the breathing transmission epidemic system have been provided using the training, verification, and testing procedures to reduce the mean square error. Moreover, the exactness and capability of the stochastic SCGGNNs are approved through error histograms, regression values, correlation tests, and state transitions.

## Introduction

An epidemic is a widespread infectious disease affecting more than one region of the world. The last century has been most notably plagued by different viral infections causing an epidemic, Middle East Respiratory Syndrome (MERS) in Saudi Arabia, Severe Acute Respiratory Syndrome (SARS), and recently coronavirus^1^. According to the World Health Organization (WHO), more than 8,000 SARS and 2200 confirmed MERS cases have been reported in 26 countries^2,3^. MERS and SARS were assumed to have fast clearance due to short incubation and transmission periods. MERS was said to have decreased relative infectivity; thus, a significantly smaller number of people were infected than SARS^4^. Recent research revealed that SARS was transmitted from person to person through berating secretions and direct contact, but MERS was only transmitted through close contact^5^. Most MERS infections are caused by unsafe close contact between MERS patients and healthcare personnel in the clinical setting^6^. The greatest MERS epidemic with steeper frequency was emphasized by the secondary pattern of MERS, which has a higher spreading heterogeneity than SARS. Most SARS and MERS cases affected the healthcare personnel and patients, respectively. Poorer prognoses and increased severity were attributed to predisposing risk factors and underlying comorbid chronic conditions in the patient population in MERS cases^7^.

Several viruses produce breathing disorders, most notably respiratory syncytial, parainfluenza, and influenza viruses. The influenza viruses cause the most common respiratory illnesses, grouped into Types *A* and *B*^8^. Every year, influenza outbreaks are spread through contact with respiratory droplets and usually cause upper respiratory tract infections affecting the nose, pharynx, and throat. The recovery rate of influenza is high and self-limited, generally requiring no treatment^9^. Ebola, Flu A/B, and SARS are easily transmissible and usually prevented with vaccinations. Various influenza virus strains infect humans, such as H7N3, H5N1, H7N7, H9N2, and H7N9.

Most epidemiological models indicate a nonlinear autonomous ordinary differential system, like HIV, SIR, and influenza disease models^10–12^. MERS and SARS transmission dynamics are also modeled mathematically^13,14^. The mathematical models help compare infection control treatments and promote a more profound knowledge of their epidemiology. This is due to the explicit form depicted in how-to link tracking and instructions control methods used to evaluate the outbreaks^15^.

**Figure 1:**
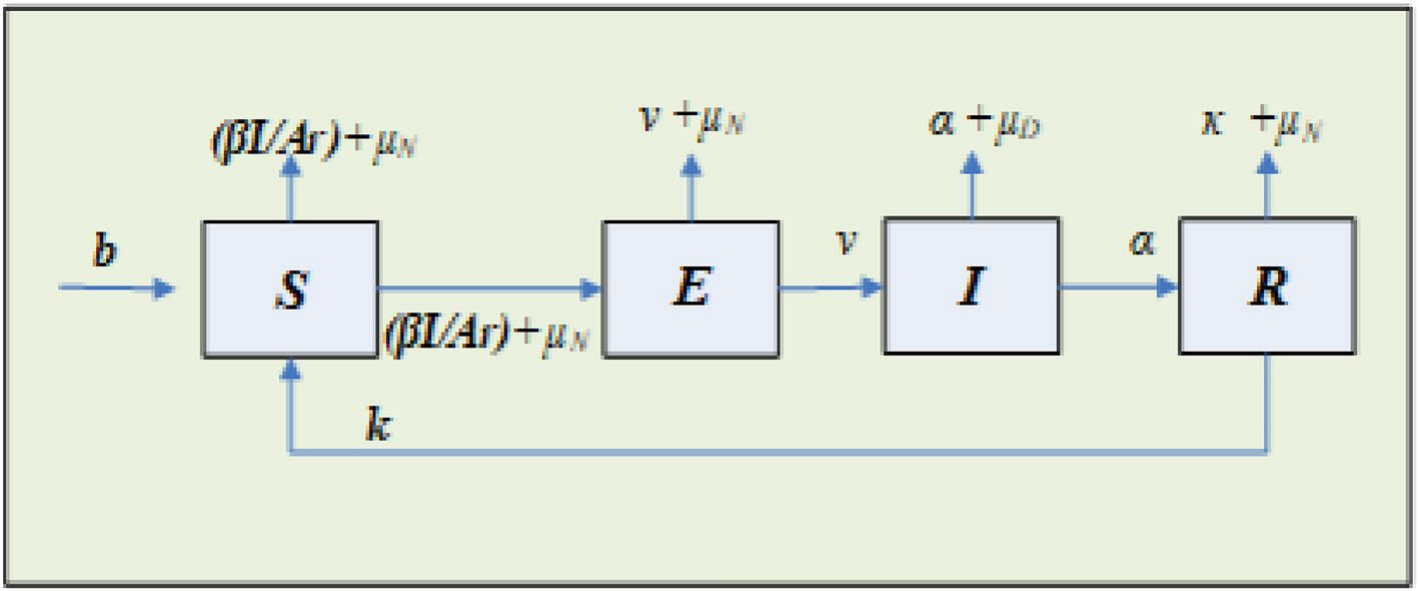
Graphical depictions of the nonlinear breathing transmission epidemic systems.

The numerical simulations of the nonlinear breathing transmission epidemic system have been presented with the stochastic scale conjugate gradient neural networks (SCGGNNs) procedures. These procedures have been recently exploited in numerous applications, e.g., functional differential systems, ocean engineering models, economic/environmental models, and thermal explosion models^16–20^. Considering these valuable stochastic submissions, the authors are motivated to present the numerical solutions to the breathing epidemic system. The classification of this model is introduced into four dynamics: susceptible, exposed, infected, and recovered, which is mathematically given as^21^:

1 $$\left\{ \begin{gathered} \frac{dS(\tau )}{{d\tau }} = b - \left( {\mu_{N} + \beta \frac{I(\tau )}{{Ar}}} \right)S(\tau ) + kR(\tau ), \hfill \\ \frac{dE(\tau )}{{d\tau }} = \beta \frac{I(\tau )}{{Ar}}S(\tau ) - (\upsilon + \mu_{N} )E(\tau ), \hfill \\ \frac{dI(\tau )}{{d\tau }} = \upsilon E(\tau ) - \left( {\mu_{D} + \alpha } \right)I(\tau ), \hfill \\ \frac{dR(\tau )}{{d\tau }} = \alpha I(\tau ) - \left( {\kappa + \mu_{N} } \right)R(\tau ), \hfill \\ \end{gathered} \right.$$

where *b* shows the rate of recruitment into the susceptible population, $\mu_{N}$ represents the natural death rate, $\beta$ is the disease transmission probability, $Ar$ shows the actual risk population, $k$ indicates the recovered rate that returns to the susceptible population based on the immunity loss, $\upsilon$ signifies the seroconversion rate, $\alpha$ denotes the recovered rate and $\mu_{D}$ expresses the death rate of disease induced. Figure 1 presents the graphical illustrations based on the nonlinear breathing transmission epidemic model presented in system (1).

The nonlinear breathing transmission epidemic model has never been solved using the proposed stochastic operators. Some more novel features of the current work are highlighted as:

- The proposed stochastic procedure successfully solves the nonlinear breathing transmission epidemic system.
- Three different values of the disease transmission probability ($\beta$) based on the breathing transmission epidemic system are employed to validate the scheme's performance.
- The SCGGNNs' accuracy is performed by matching the reference and calculated solutions.
- The comparative evaluation using the error histograms (EHs) and correlation/regression are used to evaluate metrics to demonstrate the competence of SCGGNNs.

The organization of the remaining parts of the paper is presented as Methodology in Section II, numerical solutions in Section III, and the conclusions are shown in the last Section.

## Methodology

This section presents the proposed stochastic procedure based on SCGGNNs for the breathing transmission epidemic model. The graphical procedures and the execution performances of the stochastic solvers are also presented.

### Neural network

**Figure 2:**
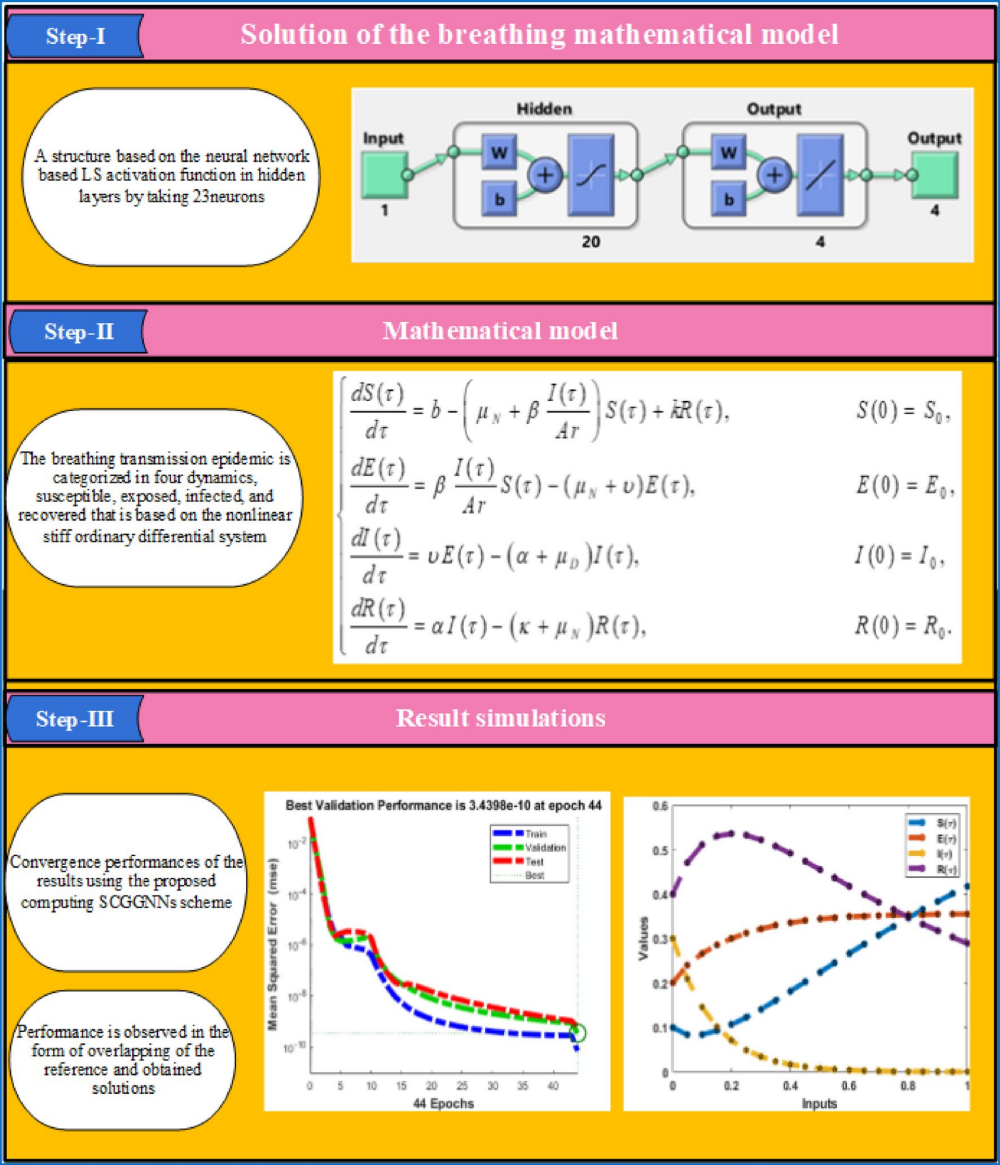
Mathematical model, layer constructions, neural structure, and result performances of the breathing transmission epidemic system.

This Section provides the structure of a neural network by taking 20 numbers of neurons in the hidden layers. An activation function based on the log-sigmoid (LS) is applied in the hidden layers, mathematically given as:

2 $$\left[ \begin{gathered} u_{1} \hfill \\ u_{2} \hfill \\ u_{3} \hfill \\ \begin{array}{*{20}c} . \\ \begin{gathered} . \hfill \\ . \hfill \\ \end{gathered} \\ {u_{20} } \\ \end{array} \hfill \\ \end{gathered} \right] = L\left( {\left[ \begin{gathered} w_{1,1} \hfill \\ w_{1,2} \hfill \\ w_{1,3} \hfill \\ \begin{array}{*{20}c} . \\ \begin{gathered} . \hfill \\ . \hfill \\ \end{gathered} \\ {w_{1,13} } \\ \end{array} \hfill \\ \end{gathered} \right][\tau ] + \left[ \begin{gathered} b_{1,1} \hfill \\ b_{1,2} \hfill \\ b_{1,3} \hfill \\ \begin{array}{*{20}c} . \\ \begin{gathered} . \hfill \\ . \hfill \\ \end{gathered} \\ {b_{1,13} } \\ \end{array} \hfill \\ \end{gathered} \right]} \right)$$

3 $$\left[ {\begin{array}{*{20}c} {S(\tau )} \\ {E(\tau )} \\ {I(\tau )} \\ {R(\tau )} \\ \end{array} } \right] = L\left( {\left[ \begin{gathered} \begin{array}{*{20}c} {\omega_{1,1} } & {\omega_{2,1} } & {\omega_{3,1} } & . & . & . & {\omega_{20,1} } \\ {\omega_{1,2} } & {\omega_{2,2} } & {\omega_{3,2} } & . & . & . & {\omega_{20,2} } \\ {\omega_{1,3} } & {\omega_{2,3} } & {\omega_{3,3} } & . & . & . & {\omega_{20,3} } \\ \end{array} \hfill \\ \omega_{1,4} \,\,\,\,\,\omega_{2,4} \,\,\,\,\,\,\omega_{3,4} \,\,\,\,\,.\,\,\,\,\,.\,\,\,\,\,\,.\,\,\,\,\,\omega_{20,4} \hfill \\ \end{gathered} \right]\left[ \begin{gathered} u_{1} \hfill \\ u_{2} \hfill \\ u_{3} \hfill \\ \begin{array}{*{20}c} . \\ \begin{gathered} . \hfill \\ . \hfill \\ \end{gathered} \\ {u_{20} } \\ \end{array} \hfill \\ \end{gathered} \right] + \left[ \begin{gathered} \begin{array}{*{20}c} {b_{2,1} } \\ {b_{2,2} } \\ {b_{2,3} } \\ \end{array} \hfill \\ b_{2,4} \hfill \\ \end{gathered} \right]} \right),$$

where $\omega$ shows the weights in hidden layers, *b* is the bias, while $S(\tau )$, $E(\tau )$, $I(\tau )$ and $R(\tau )$ are the outputs, and *L* represents the LS activation function, mathematically given as^20–24^:

4 $$L(x) = \frac{1}{{1 + e^{{ - x}} }},\;{\text{where}}\;x = b + \sum\limits_{{i = 1}}^{r} {\left( {w_{i} \tau _{i} } \right)}$$

where *r* indicates the neurons. Figure 2 represents the different steps to solve the mathematical model. Construction of the neural network by taking 20 neurons is provided in 1st part of Fig. 2, the mathematical formulations are shown in 2nd half of Fig. 2, third portion of Fig. 2 represents the obtained performances of the results.

**Figure 3:**
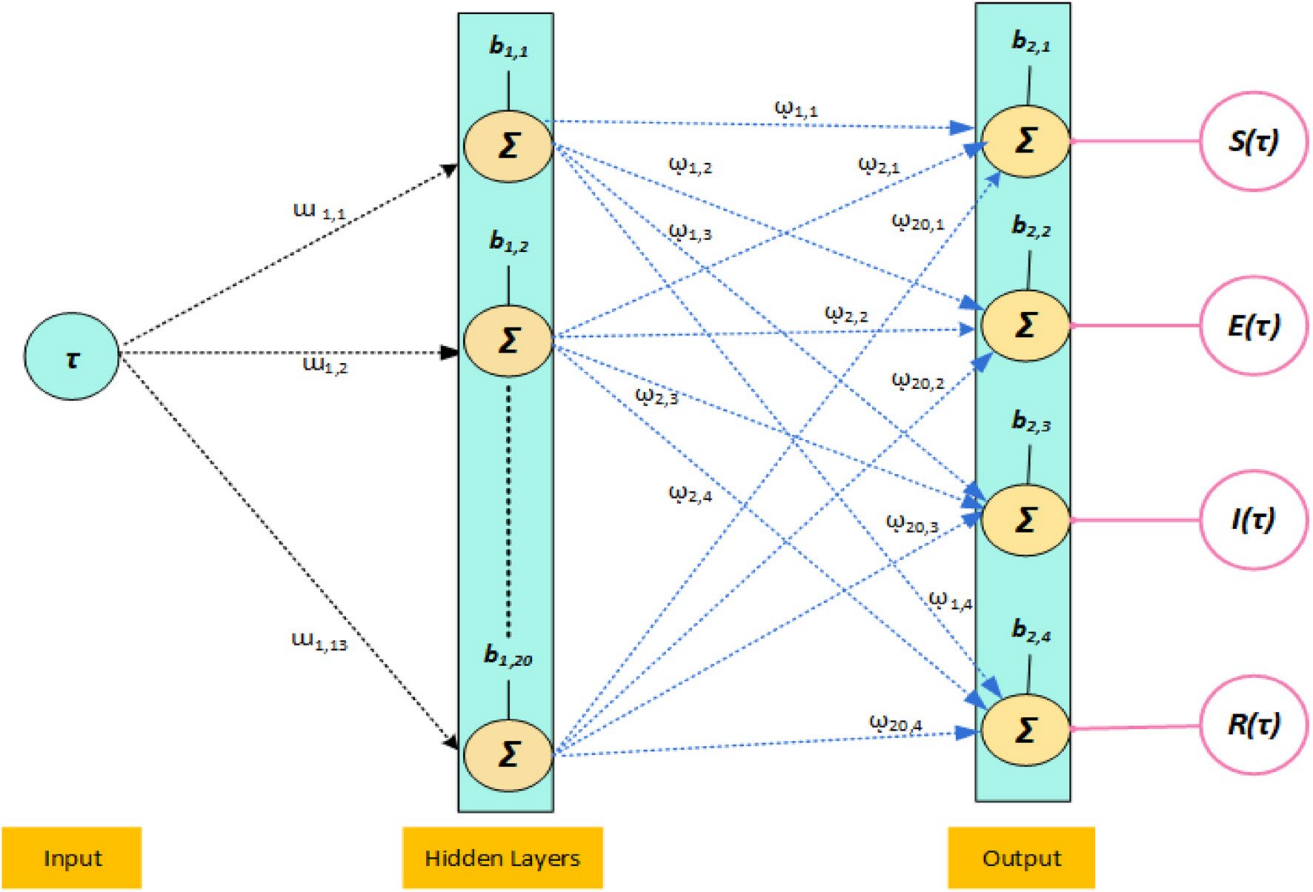
A layers structure to solve the breathing transmission epidemic system.

Figure 3 represents the structure of different layers for solving the nonlinear dynamics based on the breathing transmission epidemic system. The layered structure has been used in numerous areas, including design, communication, and technology. The process of layers effectively involves breaking down complicated processes into interconnected, manageable, and distinct components or levels. The construction of the layers-based output/input and hidden layers using twenty neurons is also presented.

**Figure 4:**
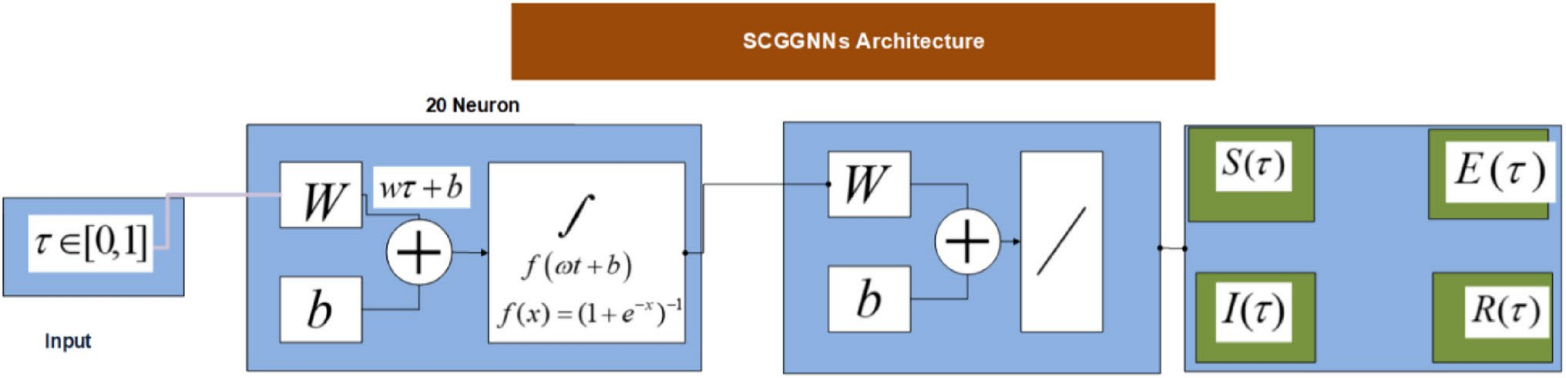
A design of layer structure for solving the breathing transmission epidemic system.

Figure 4 describes the neural network procedure using the LS activation function in the hidden layers for solving the breathing transmission epidemic system using the stochastic performances of SCGGNNs. The mathematical LS function serves as an activation function in neural networks and machine learning. It can compress large inputs to small positive values that are close to zero, while it keeps large negative to larger negative values approaching negative infinity. This is particularly useful in certain computations where numerical uncertainty is a concern, especially when dealing with extreme values.

## Numerical results

The current part of this study shows the mathematical structure of the nonlinear breathing epidemic model. Three cases based on the disease transmission probability ($\beta$) are taken as 1.1, 4.1 and 7.1, while other values are $b = 0.061,$ $\alpha = 7.222$, $\upsilon = 0.004107$, $Ar = 0.2$, $\kappa = 0.95,$ $\mu_{N} = 0.000024,$ and $\mu_{D} = 0.00000088$ including initial conditions 0.1. 0.2, 0.3 and 0.4.

**Figure 5:**
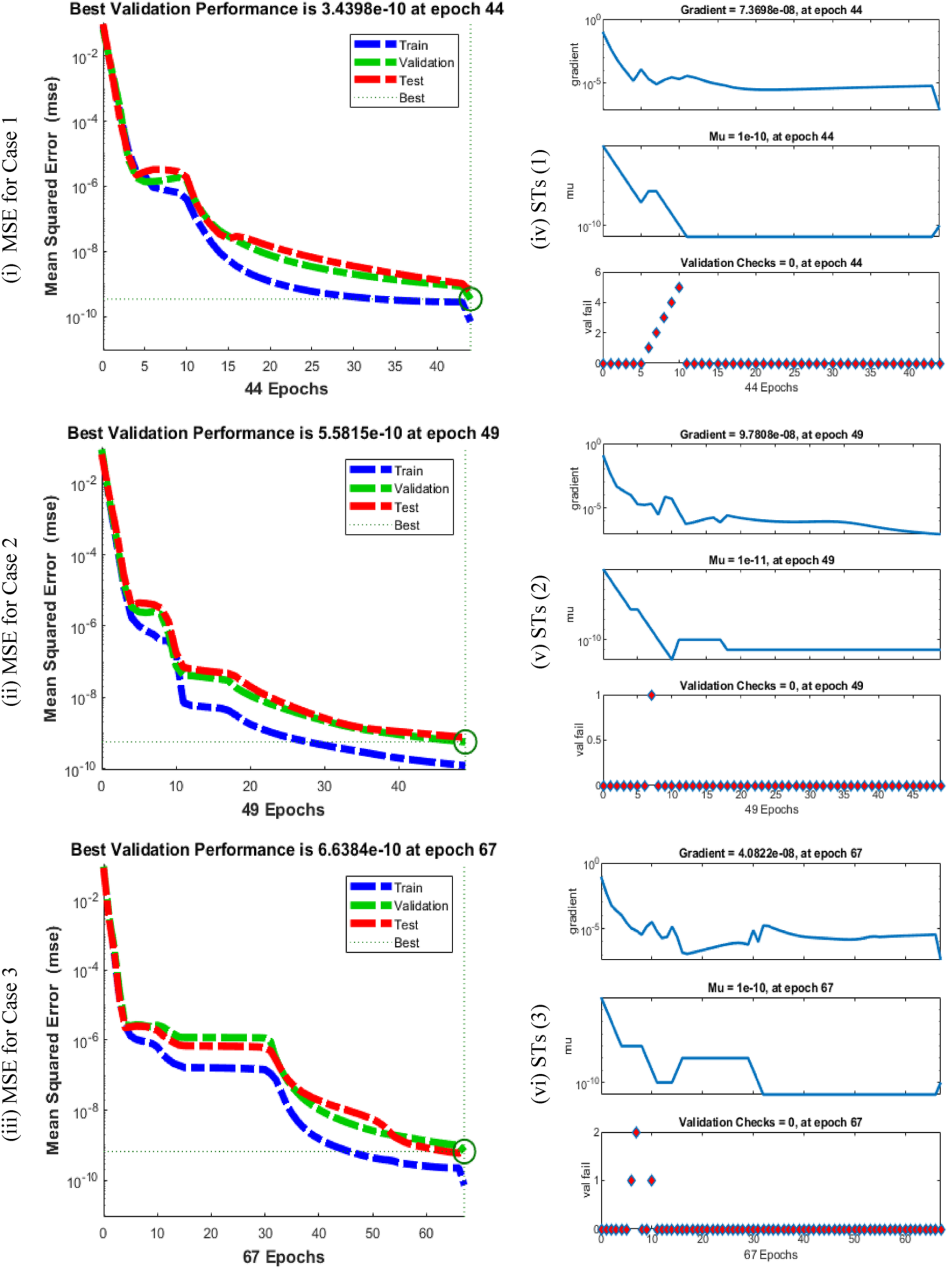
MSE tests and STs for breathing transmission system.

**Figure 6:**
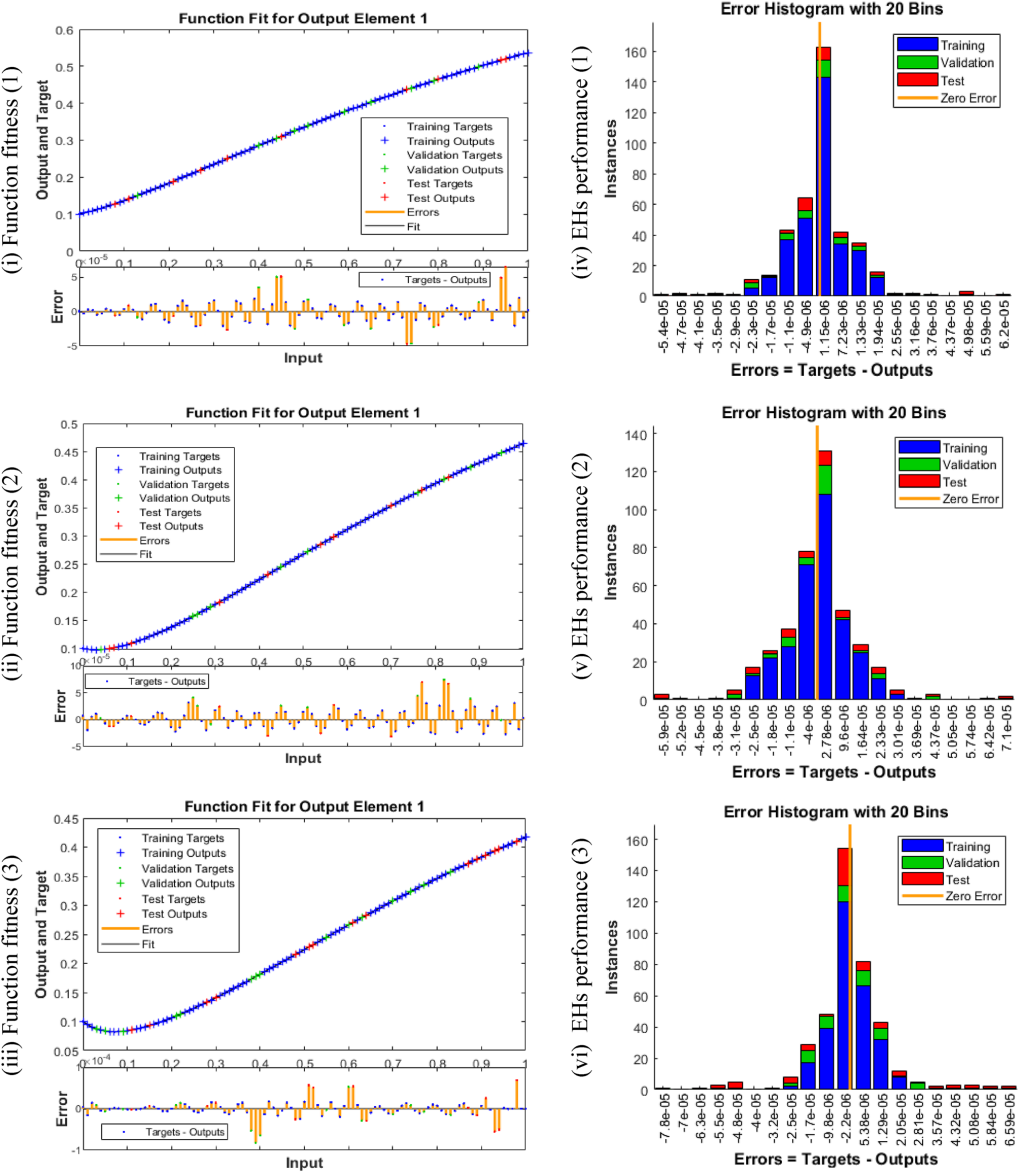
EHs and Fitness formulations for breathing transmission system.

**Figure 7:**
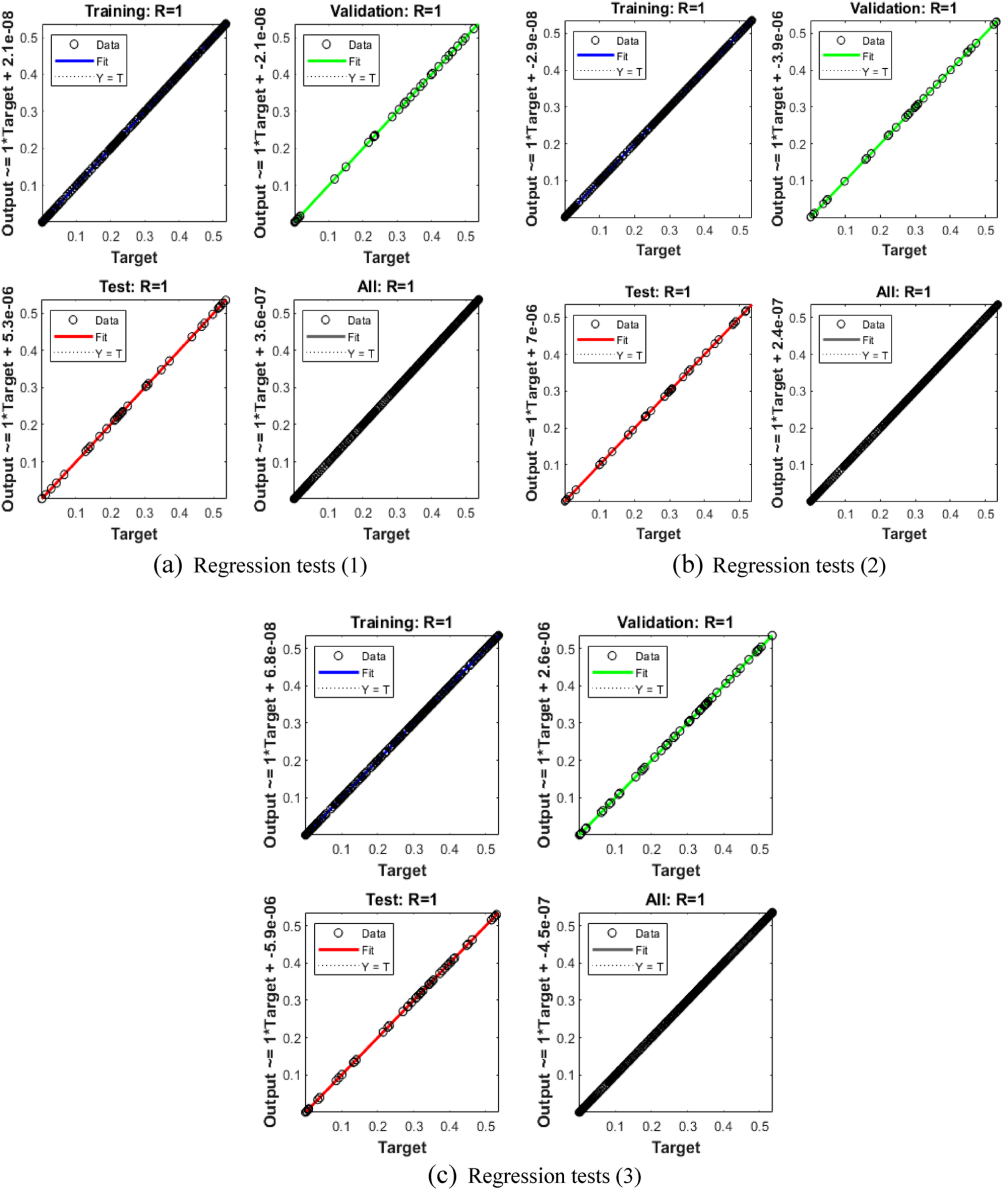
Regression tests for breathing transmission epidemic system.

Figures 5, 6 and 7 represent the performances of MSE, STs, function fitness, EHs and regression tests for three different variations of the breathing transmission epidemic system. The stochastic SCGGNNs procedures have been presented together with the LS activation function and twenty numbers of hidden neurons. Figure 5i to iii indicates the best mean square error (MSE) based on the best validation values along with Epochs. Whereas the values of Mu, gradient, and authentication checks in terms of STs are illustrated in Fig. 5 (iv-vi). The best validation values for solving the nonlinear breathing transmission epidemic model are calculated at epochs 44, 49, and 67, which are given as 3.4398 × 10^–10^, 5.5815 × 10^–10^, and 6.6384 × 10^–10^, respectively. In the process of model training and machine learning, the purpose is to design a model that can be used to generalize the hidden data. The performance of validation can also be measured using metrics like MSE, which indicates the performance of the model on unseen and new data. The perception of best validation performances in the perspective of MSE is associated with the hyperparameter tuning as well as the selection of the model. Gradient, Mu, and validation checks are considered significant concepts to train and optimize the models based on the neural networks. The gradient presents a vector and gives the information of each parameter that should be amended to degrade the loss or error of the model. Mu is an improvement to conventional gradient descent optimization schemes. It is utilized for faster optimization convergence and to avoid local minima. Validation checks are the fundamental training part based on the models of machine learning to confirm that the model is simplifying better to unseen or new data. In the process of training, a model is adjusted iteratively using the data of training. However, to avoid the case of overfitting which means the performance of the model is well based on the data of training and poorly performed on the new data, we need to consider its recital on the data of validation. The validation check is typically accomplished on each epoch. The gradient values of the operator have been presented as 7.3698 × 10^–08^, 9.7808 × 10^–08^, and 4.0822 × 10^–08^ for the breathing transmission epidemic model. These values represent the convergence performances of SCGGNNs for solving the breathing transmission epidemic system. The fitness values are performed in Fig. 6i to iii using the training outputs/targets, error performances, test outputs, validation targets/outputs, fitness, and test targets. The EHs based on zero error, authentication, test, and training for cases 1 to 3 of the breathing transmission epidemic model have been presented in Fig. 6iv-vi using the proposed SCCGNNs. The EHs are performed as 1.15 × 10^–06^ for case 1.15 × 10^–06^ and 5.38 × 10^–06^ for cases 1, 2, and 3 of the breathing transmission epidemic model. The regression analysis based on the training/testing/ validation has been illustrated in Fig. 7, and it is noticed that regression values are performed as 1 for each case (perfect model). Table 1 shows the MSE convergence using Epochs, Mu, gradient, and complexity for solving the breathing transmission epidemic model.

**Table 1:** Stochastic solvers for the breathing transmission epidemic system.

Case	MSE			Mu	Epochs	Performance	Gradient	Time
	Testing	Training	Validation					
1	5.90 × 10^–10^	6.93 × 10^–11^	3.43 × 10^–11^	1.00 × 10^–10^	44	6.93 × 10^–11^	7.37 × 10^–08^	2 Sec
2	7.46 × 10^–10^	1.20 × 10^–10^	5.58 × 10^–11^	1.00 × 10^–10^	49	1.21 × 10^–10^	9.78 × 10^–08^	2 Sec
3	6.83 × 10^–10^	7.21 × 10^–11^	6.63 × 10^–11^	1.00 × 10^–10^	67	7.22 × 10^–11^	4.08 × 10^–08^	3 Sec

**Figure 8:**
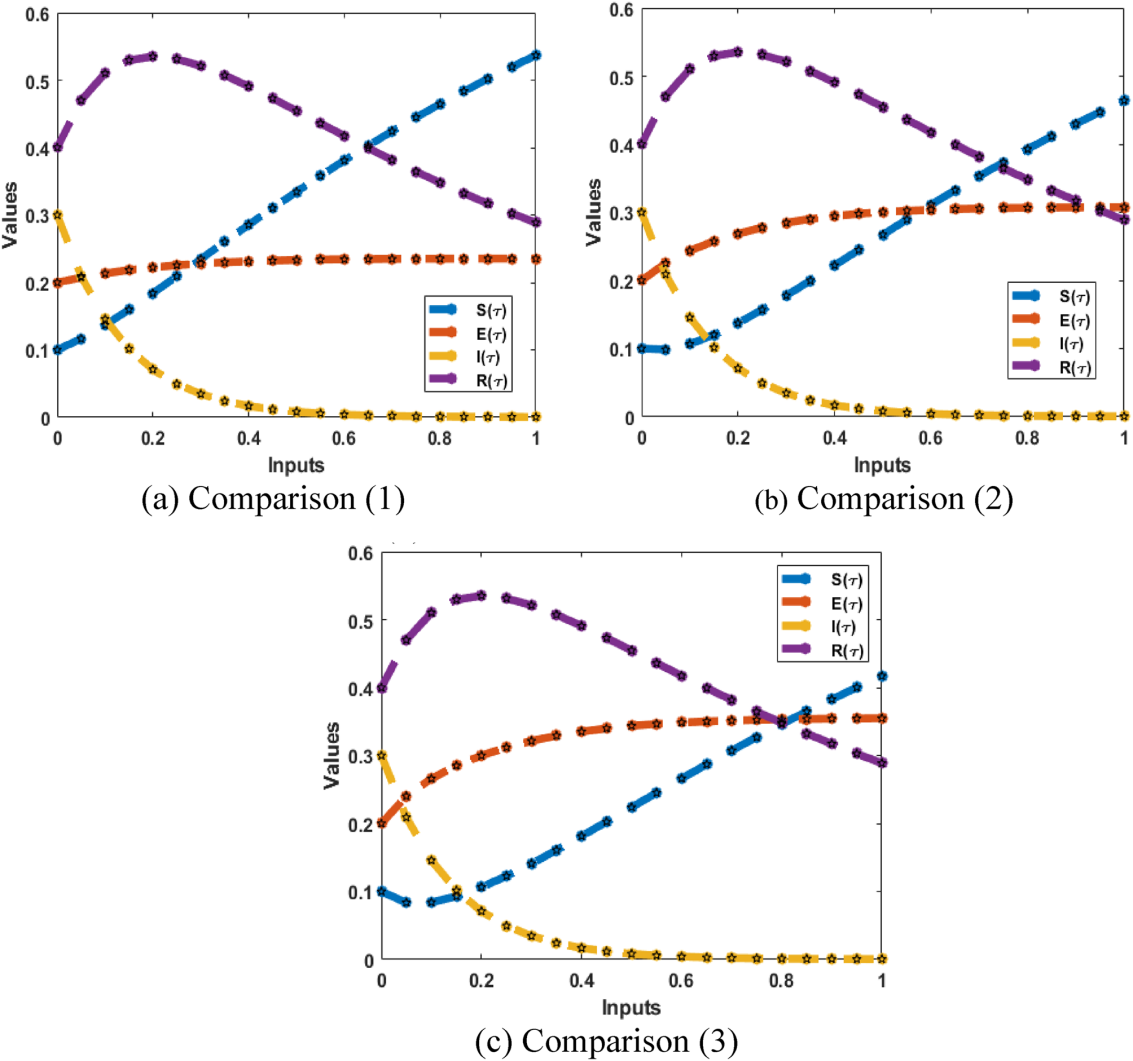
The comparison of the results performances for the breathing transmission epidemic system.

**Figure 9:**
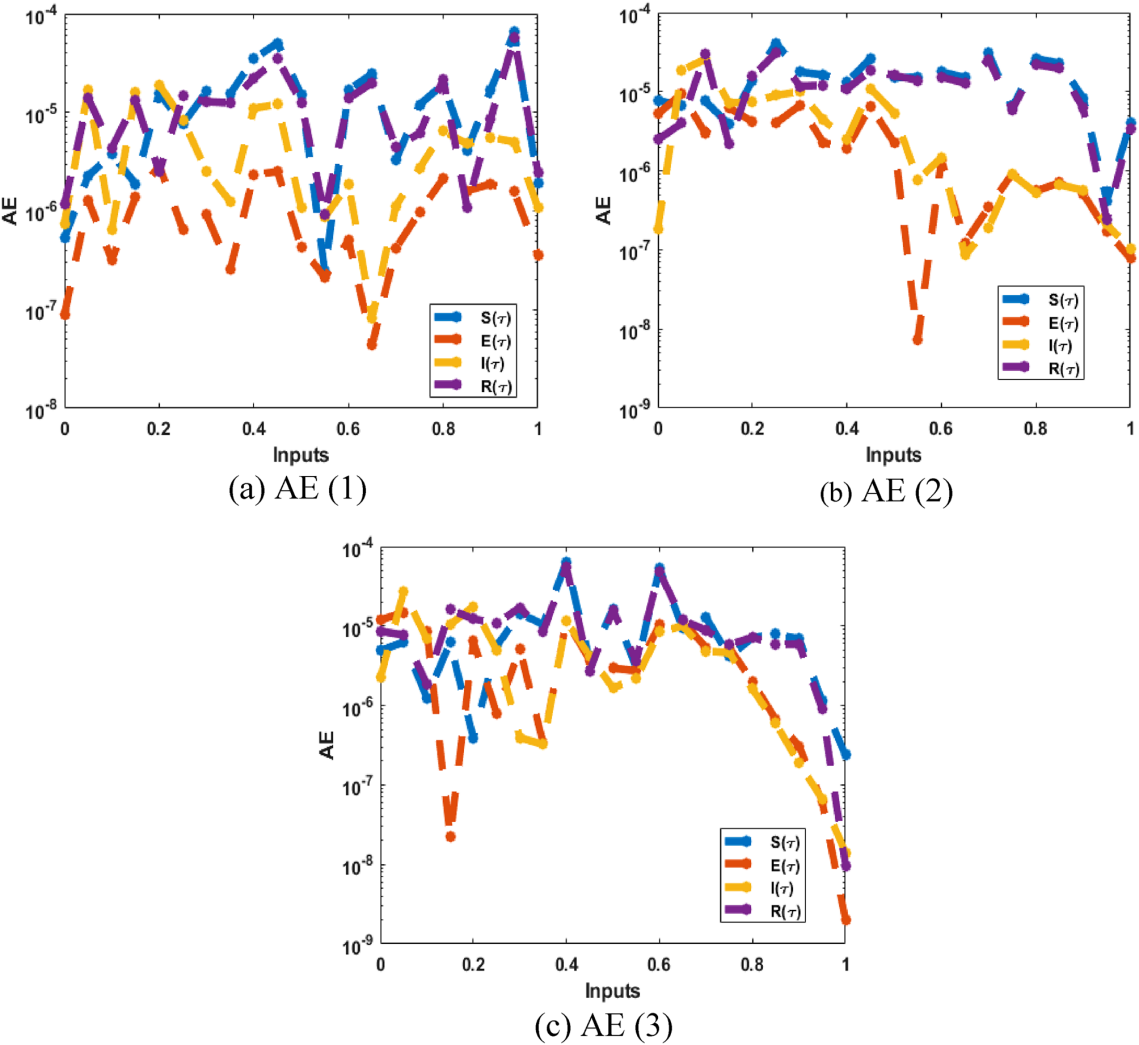
AE values for the breathing transmission epidemic system.

Figures 8 and 9 illustrate the comparison plots and AE for solving the breathing transmission epidemic mathematical model. The preciseness of the scheme is observed by overlapping the proposed and reference solutions. Moreover, the negligible AE enhances the accurateness of the procedure for solving the breathing transmission epidemic system.

## Conclusions

The numerical results of the nonlinear breathing transmission epidemic system by applying the proposed stochastic scale conjugate gradient neural network process have been presented in this study. The nonlinear form of the breathing transmission epidemic is divided into four dynamics, susceptible, exposed, infected, and recovered. The conclusions of this study are as follows:

- The nonlinear breathing transmission epidemic system has been presented through the stochastic SCGGNNs using the LS activation function along with twenty numbers of hidden neurons.
- The obtained results of the breathing transmission epidemic system have been provided using the training, verification, and testing to reduce the MSE
- The overlapping of the results and negligible values of the AE enhances the accuracy of the stochastic SCGGNNs procedure.
- The statistical computing schemes for solving the breathing epidemic model have been presented with the data selection as authentication at 74%, training at 12%, and testing at 14%.
- The correctness and competence of the stochastic SCGGNNs have been approved by using the exhaustive analysis of correlation tests, state transitions, error histograms, and regression.
- Those models which involve nonlinearity are always difficult to solve numerically. Therefore, a stochastic computing solver based on the SCGGNNS is an alternate authentic solver to solve such stiff models.

In the future, the breathing mathematical model can be implemented in real-life scenarios with applications across different areas of healthcare, nonlinear systems, and a variety of other differential models^25–35^.

## Data Availability

The datasets generated/produced during and/or analyzed during the current study/research are available from the corresponding author upon reasonable request.
